# Towards a System for Tracking Drug Delivery Using Frequency Excited Gold Nanoparticles

**DOI:** 10.3390/s19214750

**Published:** 2019-11-01

**Authors:** Nazanin Neshatvar, Rui Damaso, Nima Seifnaraghi, Andreas Demosthenous, Richard Bayford

**Affiliations:** 1 Department of Electronic and Electrical Engineering, University College London, Torrington Place, London WC1E 7JE, UK; RB1129@live.mdx.ac.uk (R.D.); a.demosthenous@ucl.ac.uk (A.D.);; 2 Department of Natural Science, Middlesex University, The Burroughs, Hendon, London NW4 4BT, UK; n.seifnaraghi@mdx.ac.uk

**Keywords:** drug delivery, electrical impedance tomography (EIT), gold nanoparticles, power amplifier, radio frequency (RF) induction

## Abstract

Nanoparticle-based drugs are rapidly evolving to treat different conditions and have considerable potential. A new system based on the combination of electrical impedance tomography (EIT) imaging and a power amplifier with a RF coil has been developed to study the effect of gold nanoparticles (AuNPs) when excited in the MHz frequency range. We show that samples including AuNPs have a temperature increase of 1–1.5 °C due to the presence of RF excitation at 13.56 MHz which provides a higher rate of change for solutions without AuNPs. They also show more than a 50% increase in conductivity in difference imaging as the result of this excitation. The change for samples without AuNPs is 40%.

## 1. Introduction

Targeted drug delivery, whereby therapeutic agents are transported from the site of administration to specifically diseased tissues, is of prime concern in pharmaceutical research. This concept has significant potential in oncology where chemotherapeutic drugs with a narrow range between effective and toxic doses can easily compromise the efficacy of the treatment. Recent progress in pharmaceutical nanotechnology has led to the development of a variety of advanced drug delivery systems (DDS) with the capacity to transport small-molecule drugs to tumors. However, new tools are required to facilitate better customization of therapy based on the specific needs of individual patients. Recently, image-guided drug delivery, which leverages clinical imaging modalities for the guidance of DDS, has emerged as a viable strategy for enhancement of targeted, personalized drug therapies. In this drug delivery paradigm, imaging may be used to identify the target and non-target anatomy or for screening, planning, monitoring, and post-procedural assessment of treatment outcome [1,2].

A novel approach to tracking nanoparticles is based on the insight that nanoparticles inside or on a part of the body of a mammal can be stimulated to generate a temperature differential within the part of the body, and this temperature differential can be detected by electrical impedance tomography (EIT). EIT is a non-invasive, non-ionizing imaging modality that is capable of functioning at high frame rates while providing good temporal resolution [3]. As an imaging modality, EIT estimates an anatomical image of the distribution of conductivity properties within a body by using current injection and voltage measurements applied at electrodes on the body surface [3]. The analysis of the sensitivity of the measured voltages for a given conductivity distribution within the body is called the forward problem; whereas image reconstruction is considered an inverse problem that calculates an estimate of the distribution of internal properties that are consistent with the measurements [3]. Using difference EIT imaging would enable estimation of the location or presence of the nanoparticles in the human body due to small changes (<1 °C) of temperature causing an impedance change.

Imaging gold nanoparticles (AuNPs) of size <2 nm or even smaller in the human body has not been possible yet; only limited work has been achieved in lab-based technologies like photo thermic microscopes or transmission electron microscopy TEM. As an example, of small size AuNPs, gold nanoclusters (Au NCs) could be used as the dual-modality contrast agent for both fluorescent and X-ray imaging [4]. Other imaging modalities using AuNPs include photoacoustic imaging that provides functional information regarding the cellular and molecular signatures of tissue by using AuNPs as contrast agents [5].

According to Nordebo et al. [6] biological tissues can be heated quickly and selectively using AuNPs subjected to a strong electromagnetic field, for example, at 13.56 MHz excitation frequency. If this thermal gradient is controlled in a way that elevates the temperature of the medium of the AuNPs by just a few degrees Celsius, it would permit the enhancement of EIT without destroying the cells [6,7].

A remote thermal activation of AuNPs can be achieved by using a radio frequency (RF) power amplifier. This is a non-invasive and ionizing radiation method that links the excitation frequency of the power amplifier to the size of the AuNPs [8]. The RF absorption of AuNPs at 13.56 MHz depends on several factors including AuNP size, charge of the ligand shell, aggregation state, and presence of electrolytes [9]. According to McCoy et al. [10] the generated heat from <2.5 nm oxidized AuNPs is through interactions of the spin magnetic moment with the oscillating magnetic field from a RF device. Sassaroli et al. [10] in another study described how the electrophoretic motion of charged AuNPs acted upon by a time-dependent electric field can be a key factor for the increased RF absorption observed in dilute solutions of AuNPs. Although two heating models have emerged, neither the electrophoretic mechanism nor the magnetic mechanism has been comprehensively evaluated, including the possibility that both could be operating in some scenarios [7,10,11].

The AuNPs under investigation are 5 nm in diameter so they can pass blood-brain barrier and primate blood vessels, and be dialyzed in the kidney allowing unwanted particles to be flushed out of the body [12,13,14].

Since RF can penetrate more easily inside tissues than optical radiation, the ability to produce deep localized heating through the interaction of RF and AuNPs could have a significant impact in localized drug delivery and cancer treatment [14]. According to [10], deeper penetration of RF radiation into biological tissue (20–200 cm) is accomplished by the use of MHz frequencies, while scattering and attenuation of near-infrared (NIR) facilitates hyperthermia for the treatment of tumors that reside <10 cm under the skin [10]. Another study by Kanwar et al. [15] emphasizes that thermal therapy with NIR energy is limited to the treatment of superficial tumors of less than 1–2 cm deep due to the significant attenuation of NIR light by biological tissues.

This paper presents an investigation into the effect of AuNPs when they are excited at 13.56 MHz frequency for the purpose of imaging with EIT. The excitation of the AuNPs will result in a faster rate of change as an increase in temperature (°C) with respect to time (minutes) when compared to samples without AuNPs. This can be seen with EIT as the variation of conductivity as a contrast agent or a faster rate of change of conductivity.

The rest of the paper is organized as follows. Section 2 explains preparation and analysis of the different AuNPs used followed by the design details of the class E RF power amplifier (PA). In Section 3, measurement results for different types of AuNP and their corresponding images when activated by the PA are analyzed. Section 4 concludes the outcome of different tomography images.

## 2. Materials and Methods

### 2.1. Preparation and Analysis of AuNPs

Two commercially available AuNPs, Sigma Aldrich (SA) and Cline Scientific (CS), were used in the experiments. SA (Product number-752568) has a core size of 5 nm diameter with hydrodynamic diameter of 9 nm as reported by the supplier. The concentration is 5.47 × 10^13^ particles/mL with the composition of Tannic acid stabilized gold and optical density (OD) of 1 [16]. CA (Product number-NP01-0051010) has a core size of 5 nm diameter and concentration of 0.06 mg/mL mass concentration reported by the supplier. The composition is Citrate stabilized gold with OD of 1 [17]. For both AuNPs, hydrodynamic diameter and OD (via DLS and UV-vis respectively) were verified and confirmed with those provided in datasheets by the supplier (available on the supplier webpage). The AuNPs were concentrated and purified with Amicon centrifugal filter units (Amicon^®^ Ultra-15, cut-off 10 kDa, Merck, UK), and re-suspended in phosphate buffered saline (PBS; DPBS 1X Gibco™, ThermoFisher, UK, product code 14190169, measured pH of 7.17). The filtration process was repeated several times.

### 2.2. RF Heating System

**Figure 1:**
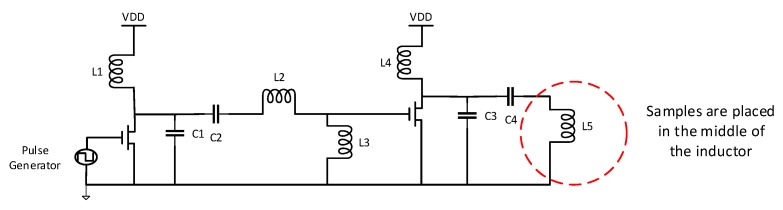
Schematic of two cascaded PAs with inductive load operating at 13.56 MHz.

A class E PA was constructed to deliver 13.56 MHz via a RF coil to excite the AuNPs samples. Class E power amplifier uses a transistor operating as a switch. The voltage-current product is low throughout the RF period during the on state of the switch and the voltage is nearly zero when high current is flowing. Thus, the transistor acts as a low resistance closed switch. During the off state of the switch the current is zero when there is high voltage, that is, the transistor acts as an open switch during the off part of the RF period [18]. The schematic of the PA is shown in Figure 1. It comprises two cascaded class E stages. The first amplifier is a drive stage followed by a second stage to achieve high power. The second amplifier is required as the input capacitance of the power MOSFET (IRLR 2703) with the 50 Ω of the source resistance would create a lowpass filter that blocks the 13.56 MHz frequency. The first amplifier alone with power MOSFET (IRLML 2030) cannot provide sufficient power (maximum achievable limit is ~1 W). The component values for the LC resonating load network are calculated based on Equations (1)–(4) where *P* is the desired power and *Q_L_* is the quality factor [18]. *L*_1,4_ are large chuck inductors to block AC signal while allowing DC to pass. The inductor *L*_3_ is needed to cancel the resonating effect of the input capacitor of the MOSFET.

(1) $$R=\left[\frac{{\left(V_{DD}-V_{o}\right)}^{2}}{P}\right]0.576801\left[1-\frac{0.4143}{Q_{L}}-\frac{0.5775}{Q_{L}^{2}}-\frac{0.2059}{Q_{L}^{3}}\right]$$

(2) $$C_{1,3}=\frac{1}{2\pi fR\left[\frac{\pi^{2}}{4}+1\right]\frac{\pi }{2}}\left[0.9986+\frac{0.9142}{Q_{L}}-\frac{1.0317}{Q_{L}^{2}}\right]+\frac{0.6}{{\left(2\pi f\right)}^{2}L_{1}}$$

(3) $$C_{2,4}=\frac{1}{2\pi fR}\left[\frac{1}{Q_{L}-0.1048}\right]\left[1.0012+\frac{1.0146}{Q_{L}-1.7879}\right]-\frac{0.2}{{\left(2\pi f\right)}^{2}L_{1}}$$

(4) $$L_{2,5}=\frac{Q_{L}R}{2\pi f}$$

The component values are given in Table 1. The resistive load of the conventional first order class E amplifier is designed to be approximately 1 Ω. It can be represented by the parasitic series resistor of the inductor which acts as the load. In this way a magnetic field is generated by a coil of several turns. A uniform magnetic field is achieved in the central part of the coil where the samples are placed. Inductor *L*_5_ is designed as a solenoid coil based on Equation (5).

(5) $$L=\frac{\mu_{o}\pi r^{2}n^{2}k}{l}$$

where *µ*_o_ is the permeability of free space, *r* is the radius of the coil, *n* is the number of turns, *k* is the Nagaoka’s coefficient [19] and *l* is the length of the coil.

In order to verify the amplifier power output, the impedance of the coil when 1 mL of AuNP solution was placed in the center of L5 was measured by a Wayne Kerr 6500B Series Impedance Analyzer to be │Z│= 130 Ω. This value is equivalent to a series combination of the inductor and resistor in parallel with the capacitor. The power at the coil is given by $\frac{V_{out,p}^{2}}{\left|Z\right|}≅8\;W$. The efficiency of the PA is not of the prime concern as the power transferred to AuNPs is very small.

**Figure 2:**
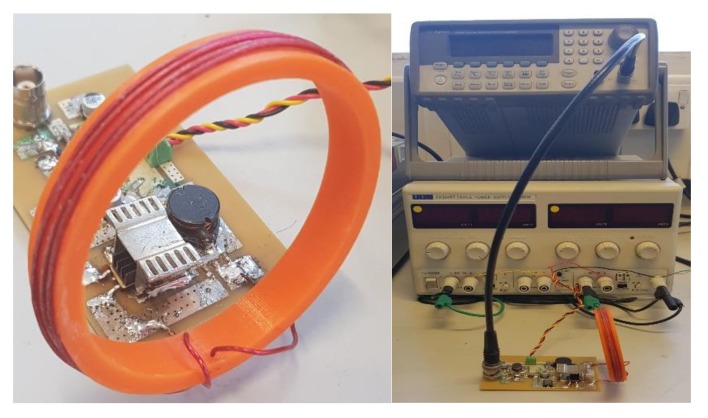
Experimental set-up for the PA and the solenoid coil as the output stage. On the left a zoomed-in image of the PA with the solenoid coil is shown, while on the right the PA, DC power supply and the function generator for providing the input signal to the PA are shown from bottom to top, respectively.

**Figure 3:**
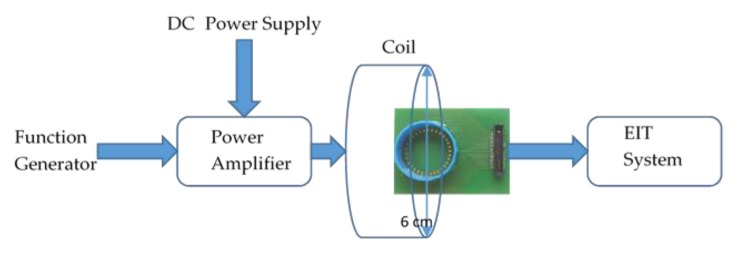
The PCB with 32 electrodes as part of the system. The dimensions of the well are 2.9 cm diameter and 1 cm height. The container material is polylactic acid (PLA).

The experimental set-up for the PA is shown in Figure 2 where a function generator provides the square wave input to the amplifier and the DC supply to power up the system. A small printed circuit board (PCB) was designed with 32 gold-plated electrodes of size 1 mm × 1.5 mm spaced evenly so that it can be interfaced with the EIT system. A well is used to cover the area where the AuNP samples are placed. Each electrode is connected to the EIT system by a ribbon cable. The PCB with the connector is shown in Figure 3.

### 2.3. EIT Imaging System

**Figure 4:**
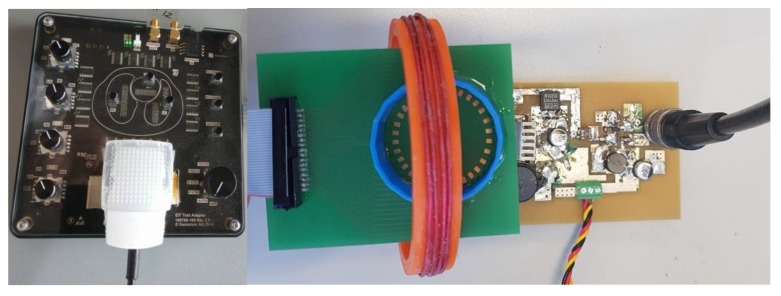
Experimental setup for imaging various AuNP solutions when activated with the designed PA operating at 13.56 MHz frequency.

The system used for EIT imaging of the AuNPs is the Pioneer set from SenTec AG [20]. It consists of an advanced interface with 32 channels, a smart SensorBeltConnector, a power supply unit and data communication documentation [21]. The system is capable of injecting programmable currents from 1 mA to 7 mA peak in the frequency range of 50 kHz to 250 kHz. It has the ability to provide different image frame rates per second (fps) [21]. The SenTec EIT monitor software (STEM) allows setting EIT parameters such as excitation frequency and injection current. It provides real-time monitoring of the signals and continuous quality checks of each one of the electrodes. The raw data can be stored for post-processing and signal analysis. Figure 4 shows the EIT setup with the PA for different AuNP measurements. The solutions are placed in the well at the middle of the small PCB with the 32 gold plated electrodes. The ribbon cable in the PCB connects the PA and AuNP solutions to the Pioneer set. All the samples where measured at 60 fps; the injected current was 7 mA peak at 250 kHz. The raw data obtained from STEM were analyzed in Matlab and EIDORS software [22] to reconstruct the images. The measurements were taken for 15 min; during the first 2 min the PA was off. The images at this time were used as the reference. Then the PA was turned on for 15 min while continuous live monitoring of the samples was performed.

## 3. Results and Discussion

### 3.1. RF Heating Data of Stuck of AuNPs

**Figure 5:**
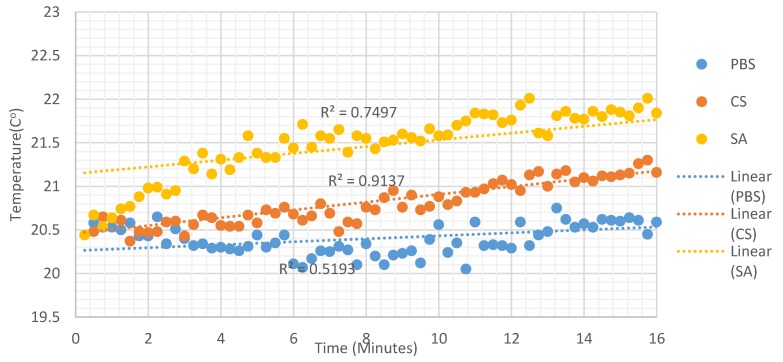
Temperature variations over 15 min of different AuNPs from the time the PA was turned on.

A Luxtron 812 fiber optic thermometer was used to measure the temperature variation of different AuNP solutions over time as a result of the heat generated from the magnetic field of the PA coil which excites the AuNPs at the resonant frequency of 13.56 MHz. Luxtron 812 fiber optic temperature monitoring is highly stable and calibrated to ± 0.2 °C within 20 °C of calibration temperature [23,24]. In this experiment, two fast immersion probes that were chemically resistant and immune to electromagnetic interference, with response times 0.25 s were used [23,24]. For the measurements, the PA was off for the first 2 min and it was turned on for the remaining 15 min. The temperature probes were placed inside the solution at 0.5 cm depth and placed in the middle of the PA coil. The temperature variations of different AuNPs are shown in Figure 5.

Figure 5 shows the PBS has the smallest temperature variation in comparison to the other solutions that contained AuNPs. PBS has temperature increase of less than 0.5 °C whereas AuNPs solutions increase by 1–1.5 °C due to the presence of RF excitation at 13.56 MHz which provides a higher rate of change with respect to PBS without AuNPs where only the ions in solution are exposed to the field. The small fluctuations in each measurement is the result of variation in the ambient temperature of the lab. CS and SA AuNPs where found to provide a linear increase with time as illustrated in Figure 5.

### 3.2. EIT Imaging with AuNPs

**Figure 6:**
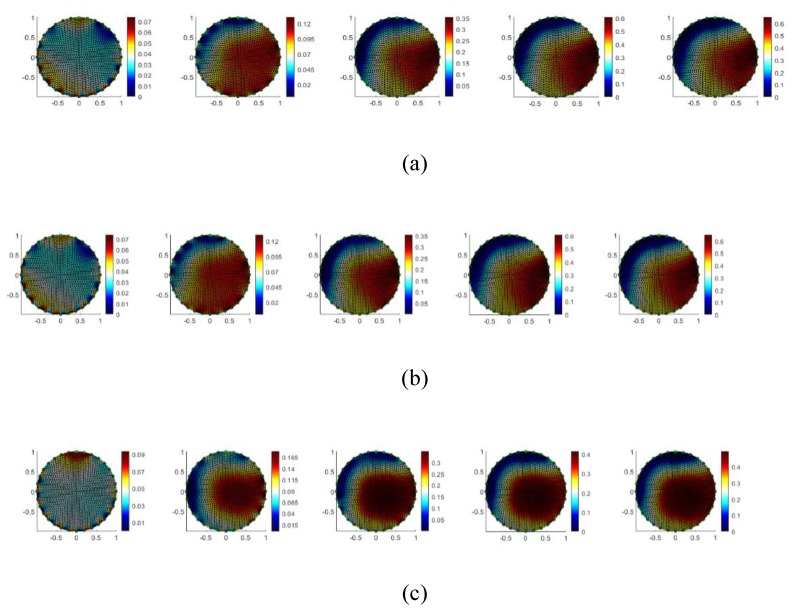
EIT images for RF excited AuNPs at 13.56 MHz: (**a**) Cline Scientific (CS) with 57.5% conductivity variation with respect to a reference based on difference imaging; (**b**) Sigma Aldrich (SA) with 54.3% conductivity variation with respect to a reference based on difference imaging; (**c**) PBS with 40.2% conductivity variation with respect to reference based on difference imaging. The axis is spatially coordinated to the transversal cross section of the well containing the sample, the dimensions are in cm. The diameter of the circle of electrodes is 2.8 cm. The color bar is % conductivity change.

The resulting images for CS, SA, and PBS are shown in Figure 6a–c respectively. The reconstruction algorithm is based on difference imaging with respect to the reference image by using GREIT algorithm [25]. CS shows a 57.7% increase in conductivity as the result of excited AuNPs at 13.56 MHz by the power amplifier and 54.3% for SA under the same condition. The results for PBS where no AuNPs are present and the ions of the solution are excited at 13.56 MHz show 40.2% increase. These results support the fact that excitation at 13.56 MHz produces a localized heat as a result of magnetic field generated by the coil. However, the rate of change for samples with AuNPs is much more than PBS where there is no AuNPs. This difference with respect to PBS is 17% and 14% for CS and SA respectively. The images are normalized and presented as a percentage change.

Due to the limitations of the EIT hardware of a maximum frequency of 250 kHz, measurements where not performed at higher frequencies. An injection current amplitude of 7 mA_p_ was used. The AuNP was added via a pipette into the middle of the solution, however, there was not a proper control on its distribution, which resulted in the red area in the images to be not exactly in the center.

## 4. Conclusions

The results demonstrate that it is possible to excite 5 nm AuNPs at 13.56 MHz and image them with EIT. It is known that 13.56 MHz could excite AuNPs at a depth of tens of centimeters which would permit tracking of AuNPs, even when they have penetrated deep inside the body thereby making them ideal for use as contrast agents to improve sensitivity of imaging inside the body or in drug delivery to various organs [10]. Their use, in conjunction with EIT measurements, proved the concept that the rate of change of conductivity in the presence of excited gold nanoparticles could be used as a diagnostic tool. The PA used in the setup was designed specifically for this size of AuNPs as the resonating load network needs to be changed for different particle dimensions. If AuNPs of other sizes are to be targeted, their resonance frequency should be studied in advance and the PA design modified accordingly. Another option would be to design a PA with variable tuning which can accommodate a specific range of frequencies based on AuNP sizes.

## Figures and Tables

**Table 1:** Component values for the PA including the inductor.

Component Name	Component Value
*VDD*	8 V
*L*_1_	270 µ
*C*_1_	390 p
*C*_2_	330 p
*L*_2_	680 n
*L*_3_	0.1 µ
*L*_4_	470 µ
*C*_3_	2.2 n
*C*_4_	57 p
*L*_5_	2.41 µ
*r*	0.03 m
*n*	4
